# QRISK3 Performance in the Assessment of Cardiovascular Risk in Patients with Inflammatory Bowel Disease

**DOI:** 10.3390/jcm10184102

**Published:** 2021-09-11

**Authors:** Marta Carrillo-Palau, Alejandro Hernández-Camba, Laura Ramos, Milagros Vela, Laura Arranz, Noemi Hernández Alvarez-Buylla, Inmaculada Alonso-Abreu, Anjara Hernández-Pérez, Manuel Hernández-Guerra, Camilo Palazuelos, Javier Llorca, Miguel Á. González-Gay, Iván Ferraz-Amaro

**Affiliations:** 1 Division of Gastroenterology, Hospital Universitario de Canarias, 38320 Tenerife, Spain; martacarry@yahoo.es (M.C.-P.); laura7ramos@gmail.com (L.R.); noehdz78@hotmail.com (N.H.A.-B.); macuaa@hotmail.com (I.A.-A.); anjarahdez@gmail.com (A.H.-P.); mhernand@ull.edu.es (M.H.-G.); 2 Division of Gastroenterology, Hospital Universitario de Nuestra Señora de la Candelaria, 38010 Tenerife, Spain; dr.alejandrohc@gmail.com (A.H.-C.); milvelillas@yahoo.es (M.V.); lauraarranz@gmail.com (L.A.); 3 Epidemiology and Public Health Group, IDIVAL, 39008 Santander, Spain; camilo.palazuelos@alumnos.unican.es; 4 Department of Medical Ciencies, University of Cantabria, CIBER Epidemiología y Salud Pública (CIBERESP), 39008 Santander, Spain; llorcaj@unican.es; 5 Epidemiology, Genetics and Atherosclerosis Research Group on Systemic Inflammatory Diseases, Hospital Universitario Marqués de Valdecilla, IDIVAL, 39008 Santander, Spain; miguelggay@hotmail.com; 6 Division of Rheumatology, Hospital Universitario Marqués de Valdecilla, Universidad de Cantabria, 39008 Santander, Spain; 7 Cardiovascular Pathophysiology and Genomics Research Unit, School of Physiology, Faculty of Health, Sciences, University of the Witwatersrand, Johannesburg 2000, South Africa; 8 Division of Rheumatology, Hospital Universitario de Canarias, 38320 Tenerife, Spain

**Keywords:** inflammatory bowel disease, cardiovascular disease, cardiovascular risk

## Abstract

Inflammatory bowel disease (IBD) has been described as an independent risk factor for the development of cardiovascular (CV) disease. Since the QRESEARCH risk estimator version 3 (QRISK3) calculator was recently proposed to assess CV in the general population, our objective was to compare the predictive ability of QRISK3 with that of a well-established European CV risk calculator, the Systematic Coronary Risk Assessment (SCORE), to identify the presence of subclinical carotid atherosclerosis in patients with IBD. In all, 186 patients with IBD and 178 controls were recruited. The presence of subclinical atherosclerosis was evaluated by carotid ultrasound to identify carotid plaque and the thickness of the carotid intima-media (cIMT). QRISK3 and SCORE were calculated. The relationship of QRISK3 and SCORE with each other and with the presence of subclinical carotid atherosclerosis (both carotid plaque and cIMT) was studied in patients and controls. SCORE (0.2 (interquartile range 0.1–0.9) vs. 0.4 (0.1–1.4), *p* = 0.55) and QRISK3 1.7 ((0.6–4.6) vs. 3.0 (1.0–7.8), *p* = 0.16) absolute values did not differ between patients and controls. QRISK3 and SCORE correlated equally with cIMT within both populations. However, SCORE correlation with cIMT was found to be significantly lower in patients with IBD when compared to controls (Spearman’s Rho 0.715 vs. 0.587, *p* = 0.034). Discrimination analysis of both calculators with carotid plaque was similar within both populations. Nevertheless, in patients with IBD, QRISK3 showed a trend toward a higher discrimination (QRISK3 area under the curve 0.812 (95%CI 0.748–0.875) vs. SCORE 0.790 (95%CI 0.723–0.856), *p* = 0.051). In conclusion, QRISK3 discrimination for subclinical atherosclerosis is optimal and equivalent to that of SCORE in IBD patients. However, our findings highlight the role of QRISK3 as an appropriate tool for the assessment of CV risk in patients with IBD.

## 1. Introduction

An increased risk of cardiovascular (CV) diseases that appears to be linked to chronic systemic inflammation has been described in patients with inflammatory bowel disease (IBD) [1]. Current CV risk calculators often underestimate the actual CV risk in well-established inflammatory diseases such as rheumatoid arthritis or systemic lupus erythematosus [2]. However, information on the predictive value of risk chart algorithms for assessing CV risk in patients with IBD is scarce.

The Systematic Coronary Risk Assessment (SCORE), which is recommended in the guidelines of the European Society of Cardiology on the prevention of CV diseases in clinical practice, is the most widely used calculator in Europe [3]. As in the general population, SCORE has been used to assess CV risk in patients with IBD, although the validation of this tool has not been well established in this population. The QRESEARCH risk estimator version 3 (QRISK3) calculator was recently proposed to assess CV in the general population [4]. In addition to the classic variables related to traditional CV risk factors, QRISK3 also includes a number of additional clinical items such as the presence of chronic kidney disease, migraine, glucocorticoid use, presence of inflammatory diseases such as rheumatoid arthritis or systemic lupus erythematosus, atypical antipsychotics, severe mental illness, and erectile dysfunction. The development of this calculator, which came from a national QRESEARCH database that included 2.3 million people, has provided the best available evidence on the weight of inflammatory diseases in estimating CV risk.

To our knowledge, QRISK3 has not been tested in patients with IBD. Therefore, in the present study, we set out to determine the usefulness of QRISK3 for CV risk assessment in patients with IBD. Since the SCORE is a well-established European CV risk calculator, we compared the predictive capacities of QRISK3 and the SCORE to identify the presence of subclinical atherosclerosis in patients with IBD, which was confirmed by ultrasound evaluation of carotid intima-media (cIMT) wall thickness and assessment of carotid plaques.

## 2. Materials and Methods

### 2.1. Study Participants

This was a cross-sectional study that included 186 consecutive patients with IBD and 178 controls from two tertiary hospitals. Patients and controls were age ±5 years and sex matched. All participants were 18 years old or older. Patients with IBD had a clinical diagnosis based on confirmed clinical, endoscopic, and histological criteria during the previous 12 months. IBD patients had been diagnosed by a gastroenterologist and were periodically followed up at gastroenterology outpatient clinics. For the purpose of inclusion in the present study, IBD disease duration had to be ≥1 year. The controls were community based and recruited by general practitioners in primary health centers. Since diabetes is an equivalent of very-high CV risk, controls and patients with diabetes were excluded. Likewise, patients and controls were excluded if they had a history of myocardial infarction, angina, stroke, a glomerular filtration rate <60 mL/min/1.73 m^2^, a history of cancer, and/or any other chronic disease or evidence of active infection. The study protocol was approved by the Institutional Review Committee at Hospital Universitario de Canarias and Hospital Universitario Nuestra Señora de La Candelaria, both in Spain, and all subjects provided informed written consent (approval no. CHUC_2019_103). Research carried out with human subjects was in compliance with the Helsinki Declaration.

### 2.2. Data Collection

Surveys in IBD patients and controls were performed to assess CV risk factors and medication. Hypertension was defined as a systolic or a diastolic blood pressure higher than, respectively, 140 and 90 mmHg, and according to the 2018 ESC/ESH Guidelines for the management of arterial hypertension [5] Disease activity in Crohn’s disease was assessed using the Crohn’s Disease Activity Index (CDAI) and the Harvey–Bradshaw Index (HBI) [6]. Disease activity in ulcerative colitis was calculated through the partial Mayo Clinic score [7]. The Systematic Coronary Risk Evaluation (SCORE) was assessed as previously described [3]. QRISK3 was estimated using the R package developed by Yan Li et al. [8]. Because all patients belong to a Spanish population, the UK post code variable included in this risk calculator was handled as missing. A carotid ultrasound examination was used to assess carotid intima-media wall thickness (cIMT) in the common carotid artery and to detect focal plaques in the extracranial carotid tree in patients with IBD and controls as described elsewhere [9]. Plaque criteria were based on the Mannheim consensus [10].

### 2.3. Statistical Analysis

Demographic and clinical characteristics are shown as frequencies for binary variables. Continuous variables data are expressed as mean ± standard deviation (SD) or as a median and interquartile range (IQR) for non-normally distributed variables. Linear association between continuous variables was studied using Spearman’s Rho correlation coefficient. Comparison between these correlation coefficients was performed using the Fisher r-to-z transformation. Relations of QRISK3 and SCORE to the presence of carotid plaque were analyzed through the relation of sensitivity versus false positive frequency (1-specificity) using receiver-operating characteristic curves (ROC). A comparison of ROC curves to test the statistical significance of the difference between the areas under two dependent ROC curves (AUC) (derived from both the same and different cases) was conducted using the method of DeLong et al. [11]. All analyses used a 5% two-sided significance level and were performed using SPSS software, version 25 (IBM, Chicago, IL, USA).

## 3. Results

### 3.1. Demographic, Laboratory, and Disease-Related Data

A total of 186 non-diabetic IBD patients and 178 age ± 5 years and sex-matched controls with a mean ± SD age of 48 ± 10 and 45 ± 12 years, respectively, were included in this study. Demographic and disease-related characteristics of the participants are shown in Table 1. Body mass index (27 ± 5 vs. 26 ± 4 kg/m^2^, *p* = 0.006) and waist circumference (93 ± 12 vs. 90 ± 14 cm, *p* = 0.048) were higher in IBD patients than in controls, although the size effect of this difference was small. Whereas there were no differences in the prevalence of smoking or hypertension, patients with IBD were more commonly obese (27% vs. 12%, *p* = 0.001). No differences were found in lipid profile molecules with the exception of triglycerides that were significantly higher in patients with IBD.

In all, 68% of the patients had Crohn’s disease and 32% ulcerative colitis. The median disease duration of IBD was 14 ± 9 years. Crohn’s disease patients had mostly the ileal inflammatory type. Median CDAI score was 39 (IQR −18–85), and 89% of the patients were considered to be in clinical remission. Similarly, the Harvey–Bradshaw Index was 2 (IQR 0–4), and most of the patients (82%) were in the clinical remission category of this index. Regarding ulcerative colitis, 49% were pancolitis, and 76% of the patients had a partial Mayo score inferior to 1 point. Statin intake was not associated with IBD phenotypes in either ulcerative colitis or Crohn’s disease. Similarly, there was no difference in the percentage of patients undergoing anti-TNF therapy between the two types of IBD. In this regard, while 35% of Crohn’s disease patients underwent anti-TNF therapy, 24% of ulcerative colitis patients received these agents (*p* = 0.13). Additional information regarding disease-related data is shown in Table 1.

Concerning carotid ultrasound assessment, 33% of the IBD patients had carotid plaques compared to 25% of controls (*p* = 0.067). The average cIMT in patients and controls was 641 ± 137 mm and 603 ± 115 mm, respectively (*p* = 0.006) (Table 1).

### 3.2. QRISK3 and SCORE: Relationship between Them with Respect to Carotid Plaque and cIMT in Patients and Controls

The absolute values of QRISK3 and SCORE did not differ between patients and controls. This indicates that, according to both risk graph calculators, the CV risk in the two populations was similar.

Correlations of QRISK3 and SCORE with cIMT were high in patients and controls. Furthermore, in IBD, the comparison of Spearman’s Rho correlation coefficients showed no differences, indicating that both calculators were equally correlated with cIMT in these patients. This was also the case for controls in which correlation coefficients of the two scores with cIMT were not statistically significantly different (Table 2). When the correlation of QRISK3 with cIMT was compared in patients and controls, no significant differences were found. However, the SCORE correlation with cIMT was stronger in controls than in IBD patients (Spearman’s Rho 0.715 in controls vs. 0.587 in patients, *p* = 0.034) (Table 2).

Moreover, Spearman’s Rho correlation coefficients of cIMT to QRISK3 and SCORE did not differ between IBD types. In this sense, QRISK3 had a correlation coefficient to cIMT of 0.645 and 0.596 (*p* = 0.62) and SCORE of 0.951 and 0.955 (*p* = 0.79) in, respectively, ulcerative colitis and Crohn’s disease.

The AUCs for the presence of carotid plaque in patients and controls were high. As with the cIMT, the AUCs did not differ between the two calculators in the analysis in each population. However, a trend toward greater discrimination of the carotid plaque was observed with QRISK3 than with SCORE but only in patients with IBD (QRISK3′s AUC 0.812 (95%CI 0.748–0.875) vs. SCORE’s AUC 0.790 (95%CI 0.723–0.856), *p* = 0.051) (Table 2). Similarly, QRISK3 and SCORE discrimination for plaque did not differ when calculators were compared between patients and controls. In addition, the AUC of QRISK3 and SCORE to carotid plaque was not different between ulcerative colitis and Crohn’s disease (SCORE to carotid plaque in ulcerative colitis and Crohn’s disease 0.821 and 0.768, *p* = 0.41; QRISK3 to carotid plaque in ulcerative colitis and Crohn’s disease: 0.845 and 0.767, *p* = 0.25).

**Figure 1:**
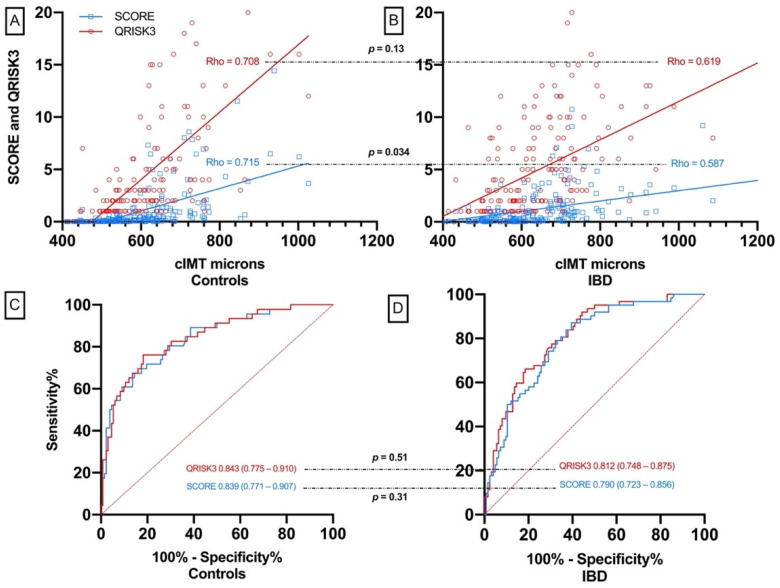
Relation of QRISK3 and SCORE to cIMT and carotid plaque in IBD patients and controls. Comparison between patients and controls are additionally shown. (**A**) relation of cIMT to SCORE and QRISK3 in controls. (**B**) relation of cIMT to SCORE and QRISK3 in IBD patients. (**C**) ROC of SCORE and QRISK3 to carotid plaque in controls. (**D**) ROC of SCORE and QRISK3 to carotid plaque in IBD patients.

A graphical representation of Table 2 is shown in Figure 1.

## 4. Discussion

Our study is the first in the literature to test the performance of two CV risk algorithms in patients with IBD. Our results show that QRISK3 may be a suitable calculator tool for the assessment of CV risk in IBD. This is based on the fact that the relationship of SCORE with cIMT was higher in patients than in controls, and that the association of QRISK3 with carotid plaque showed a trend to be greater than that of SCORE in patients with IBD.

There is clear evidence implicating high-grade inflammation as a pathway to accelerated vascular disease in the general population and in patients with chronic inflammatory diseases such as rheumatoid arthritis [12]. In this sense, systemic inflammation appears to enhance CV risk directly and indirectly via accentuation of existing risk pathways. QRISK3 is a score that includes inflammatory diseases in its calculation such as rheumatoid arthritis and systemic lupus erythematosus, but not IBD. For this reason, this calculator could better predict subclinical atherosclerosis in patients with IBD. Our data confirm previous results reported in other inflammatory diseases such as rheumatoid arthritis [13], spondyloarthropathies [14], or systemic lupus erythematosus [15]. We believe that the inflammatory load present in patients with IBD may also be responsible for this discrepancy between scores in patients and controls.

As is well known, ulcerative colitis and Crohn’s disease represent different spectra of IBD. Despite this, our results were not different when each type of IBD was analyzed separately. In this sense, the relationships of both scores with cIMT or carotid plaque did not differ between both disease types. We believe, therefore, that the findings of our study can be applied to the two forms of IBD in an equivalent manner.

It should be noted that the patients and controls in our study were not perfectly matched for CV or demographic risk factors. However, this is not strictly necessary as both the QRISK3 and SCORE calculators already adjust their values according to these factors. Furthermore, as has been shown, the absolute values of both calculators did not differ between patients and controls, thus showing that the CV risk values were similar in both populations. This implies that a comparison between both algorithms and both populations could be made without assuming the presence of confounding factors.

We recognize that our sample size can be considered relatively small for this type of study. For this reason, we believe that our findings warrant further future studies with a larger number of subjects and in a prospective design. Similarly, although most scores predict mortality or CV events, our study was carried out with subclinical carotid atherosclerosis as the outcome. This may be a limitation, but it must also be taken into account that the presence of carotid plaque has proven to be a perfect predictor of future CV events both in the general population and in patients with chronic inflammatory diseases such as rheumatoid arthritis [13,16]. Further, the study was performed in a Spanish population that is considered a low CV mortality risk country. For this reason, we believe that our findings would need to be replicated in other IBD populations.

In conclusion, our results indicate that QRISK3 calculator is useful for the CV risk assessment in patients with IBD.

## Figures and Tables

**Table 1:** Characteristics of patients with inflammatory bowel disease and controls.

	Controls	IBD Patients	
	(*n* = 178)	(*n* = 186)	*p*
Age, years	45 ± 12	48 ± 10	**0.002**
Male, *n* (%)	90 (51)	85 (46)	0.35
Body mass index, kg/m^2^	26 ± 4	27 ± 5	**0.006**
Abdominal circumference, cm	90 ± 14	93 ± 12	**0.048**
Systolic blood pressure, mmHg	125 ± 15	125 ± 19	0.77
Diastolic blood pressure, mmHg	78 ± 9	74 ± 12	**<0.001**
Cardiovascular co-morbidity			
Smoking, *n* (%)	32 (18)	36 (19)	0.14
Diabetes, *n* (%)	0 (0)	0 (0)	-
Hypertension, *n* (%)	20 (11)	31 (17)	0.14
Obesity, *n* (%)	22 (12)	50 (27)	**0.001**
Statins, *n* (%)	15 (8)	16 (9)	0.83
Analytical and lipid profile			
CRP, mg/L	0.8 (0.5–2.0)	1.8 (0.9–3.6)	**0.026**
Cholesterol, mg/dL	200 ± 33	204 ± 49	0.35
Triglycerides, mg/dL	102 ± 55	147 ± 88	**<0.001**
HDL cholesterol, mg/dL	59 ± 17	57 ± 18	0.46
LDL cholesterol, mg/dL	120 ± 31	117 ± 40	0.45
LDL:HDL cholesterol ratio	2.21 ± 0.87	1.17 ± 0.86	0.66
Non-HDL cholesterol, mg/dL	145 ± 37	146 ± 43	0.66
Atherogenic index	3.63 ± 1.06	3.77 ± 1.16	0.28
IBD related data			
Crohn’s disease, *n* (%)		127 (68)	
Ulcerative colitis, *n* (%)		59 (32)	
Disease duration since diagnosis, years		14 ± 9	
Crohn’s Disease related data, *n* (%)			
A1 below 16 years		19 (15)	
A2 between 17 and 40 years		79 (62)	
A3 above 40 years		26 (20)	
L1 ileal		55 (43)	
L2 colonic		23 (18)	
L3 ileocolonic		49 (39)	
L4 isolated upper disease		11 (9)	
B1 non-stricturing, non-penetrating		71 (56)	
B2 stricturing		45 (35)	
B3 penetrating		14 (11)	
CDAI score		39 (−18–85)	
Harvey-Bradshaw Index		2 (0–4)	
Ulcerative Colitis related data, *n* (%)			
Proctosigmoiditis		6 (10)	
Left-sided colitis		22 (37)	
Pancolitis		29 (49)	
Partial Mayo score		0 (2–4)	
Fecal calprotectin >120 mcg/g		76 (41)	
Perianal disease, *n* (%)		22 (12)	
Previous surgery, *n* (%)		54 (29)	
Oral mesalazine, *n* (%)		60 (32)	
Methotrexate, *n* (%)		21 (11)	
Azathioprine, *n* (%)		58 (31)	
Anti-TNF therapy, *n* (%)		56 (30)	
Ustekinumab, *n* (%)		8 (4)	
Vedolizumab, *n* (%)		5 (3)	
Tofacitinib, *n* (%)		4 (2)	
Carotid intima-media assessment			
Carotid plaque, *n* (%)	46 (25)	62 (33)	0.12
bilateral, *n* (%)	19 (10)	30 (16)	0.13
cIMT, microns	603 ± 115	641 ± 137	**0.006**

Data represent mean ± SD or median (interquartile range) when data were not normally distributed. BMI: body mass index; CRP: C reactive protein; LDL: low-density lipoprotein. HDL: high-density lipoprotein; TNF: tumor necrosis factor; cIMT: carotid intima-media thickness. CDAI: Crohn’s Disease Activity Index. Significant *p* values are depicted in bold.

**Table 2:** Relationship of QRISK3 and SCORE with subclinical carotid atherosclerosis in patients and controls.

SCORE		*p*
Controls	0.2 (0.1–0.9)	0.55
Patients	0.4 (0.1–1.4)	
QRISK3		
Controls	1.7 (0.6–4.6)	0.16
Patients	3.0 (1.0–7.8)	
Spearman’s Rho correlation between cIMT and QRISK3 or SCORE		
Controls		
QRISK3	0.708	**<0.001**
SCORE	0.715	**<0.001**
Difference of correlation coefficients		0.90
Patients		
QRISK3	0.619	**<0.001**
SCORE	0.587	**<0.001**
Difference of correlation coefficients		0.63
Patients vs. controls		
QRISK3 in patients vs. QRISK3 in controls		0.13
SCORE in patients vs. SCORE in controls		**0.034**
Differences in AUC for carotid plaque discrimination between calculators		
Controls		
QRISK3	0.843 (0.775–0.910)	0.67
SCORE	0.839 (0.771–0.907)	
Patients		
QRISK3	0.812 (0.748–0.875)	0.051
SCORE	0.790 (0.723–0.856)	
Patients vs. controls		
QRISK3 in patients vs. QRISK3 in controls		0.51
SCORE in patients vs. SCORE in controls		0.31

Data represent means ± SD or median (interquartile range). AUCs are expressed as area and 95% confidence interval. cIMT: carotid intima-media thickness; AUC: area under the curve. SCORE: Systematic Coronary Risk Assessment. QRISK3: QRESEARCH risk estimator version 3. Significant *p* values are depicted in bold.

## Data Availability

The data presented in this study are available on request to the corresponding author.
